# Dietary Habits and Stomach Cancer Risk in the JACC Study

**DOI:** 10.2188/jea.15.S98

**Published:** 2005-08-18

**Authors:** Noritaka Tokui, Takesumi Yoshimura, Yoshihisa Fujino, Tetsuya Mizoue, Yoshiharu Hoshiyama, Hiroshi Yatsuya, Kiyomi Sakata, Takaaki Kondo, Shogo Kikuchi, Hideaki Toyoshima, Norihiko Hayakawa, Tatsuhiko Kubo, Akiko Tamakoshi

**Affiliations:** 1 Department of Clinical Epidemiology, Institute of Industrial Ecological Sciences, University of Occupational and Environmental Health.; 2 Fukuoka Institute of Health and Environmental Sciences.; 3 Fukuoka Institute of Occupational Health.; 4 Department of Preventive Medicine, Graduate School of Medical Sciences, Kyushu University.; 5 Department of Public Health, Showa University School of Medicine.; 6 Department of Public Health/ Health Information Dynamics, Field of Social Life Science, Program in Health and Community Medicine, Nagoya University Graduate School of Medicine.; 7 Department of Public Health, Wakayama Medical University.; 8 Department of Medical Technology, Nagoya University School of Health Sciences.; 9 Department of Public Health, Aichi Medical University School of Medicine.; 10 Department of Epidemiology, Research Institute for Radiation Biology and Medicine, Hiroshima University.; 11 Department of Preventive Medicine/ Biostatistics and Medical Decision Making, Nagoya University Graduate School of Medicine.; 12 See acknowledgment for the investigators (name and affiliation) involved in the JACC Study.

**Keywords:** Diet, Prospective Studies, Stomach Neoplasms, Risk Factors, the JACC Study

## Abstract

BACKGROUND: Despite a declining incidence, stomach cancer is still a dominant cancer in Japan. The association between dietary habits and stomach cancer risk was investigated in a large prospective study in Japan.

METHODS: Data were obtained using a self-administered questionnaire from 1988 through 1990. Food frequency questionnaire was used to evaluate the consumption of 33 selected food items. Proportional hazard model was used to determine the hazard ratios (HRs) and their 95% confidence intervals (CIs) of stomach cancer for different levels of the dietary intakes.

RESULTS: A western style breakfast showed an inverse association with stomach cancer risk in males (HR=0.49, 95% CI: 0.35-0.70). Women who consumed liver three to four times per week and more than once per day had a significant increased risk, respectively (HR=2.02, 95% CI: 1.12-3.63, HR=3.16, 95% CI: 1.16-8.62 ). A clear dose-response relationship between the intake of liver and stomach cancer risk was observed. We found no association between stomach cancer mortality and the consumption of fruit such as mandarin orange, and vegetables such as carrots and spinach in both men and women. The consumption of high salt foods such as miso soup and pickles was also not significantly associated with the mortality of stomach cancer in both sexes.

CONCLUSION: This prospective study suggested that a western-style breakfast is associated with a lower risk of stomach cancer, although some differences in the association were seen between men and women.

Steady declines in incidence and mortality rates of stomach cancer have been found worldwide in recent decades.^1^ Despite a declining incidence, stomach cancer is still a dominant cancer in Japan.^2^ Previous studies indicated that environmental factors, particularly diet, play an important role in the etiology of this cancer.^3^^,^^4^ An increased risk of stomach cancer has been linked to low dietary intake of fresh fruit and vegetables and a high consumption of starch foods.^5^^-^^10^ Other reports have suggested that the risk of stomach cancer increased in a population who consumed cured or preserved fish and meat in salt or smoked.^11^^-^^13^ However, most of this epidemiologic evidence was based on studies with a case-control design. The results from case-control studies can be biased by differential recall of dietary intake due to stomach disorders. Recall of dietary intake could also have been influenced by current dietary habits. Prospective studies are less susceptible to recall bias because dietary intake is measured before the presence of a disease in known. Unfortunately, there have been a limited number of prospective studies in Japan.^14^^-^^16^ In addition, these studies have less information on lifestyle and the number of subjects was small.

Monbusho cohort study, a large-scale prospective cohort study on life style including dietary habits and cancer, began in 1988 with about 125,000 men and women. This cohort study was conducted by more than twenty universities and research institutes that were located throughout Japan. Therefore, data in this cohort would be representative of the Japanese population. We have investigated the relation between dietary intakes and mortality of stomach cancer.

## METHODS

### JACC study

The Japan Collaborative Cohort Study (JACC Study) for Evaluation of Cancer Risk sponsored by the Ministry of Education, Science, Sports and Culture of Japan (Monbusho) was established from 1988 through 1990 to investigate the relationship between various lifestyle variables and cancer mortality and incidence. The study methods have been described in detail elsewhere.^17^ In brief, this cohort consisted of 110,792 persons aged 40 -79 years who lived 52 cities, towns and villages in Japan at the beginning of the study. At baseline, the cohort members completed a self-administered questionnaire on dietary habits, smoking, alcohol use, medical history, personal and family history of cancer and demographic data. We followed the subjects from 1988 through 1999. Information on deaths among the study subjects was annually collected by examination of death certificates with permission of the Management and Coordination Agency of the Japanese Government. All baseline data and follow up data including death data were reported to the central committee office. The underlying causes of death were coded according to International Classification of Disease Revision 9 from 1988 through 1994 and after 1995, Revision 10. Stomach cancer was determined by the coding 151.0-159.0 for ICD-9 and C16.0-C16.9 for ICD-10. Informed consent was obtained from each subject.

To participate in the study, an individual’s informed consent was obtained in 36 out of 45 areas (written consent in 35 areas and oral consent in 1 area), though in 9 areas, group consent from head of the area was obtained.

### Dietary questionnaire

The dietary section of the questionnaire was a 33 item food frequency questionnaire dealing with on the usual consumption of food and beverages. Frequency consumption of 33 foods was classified into five categories (almost never, less than once or twice per month, once or twice per week, three or four times per week, or almost daily). Food items of vegetables and fruits include spinach, carrot/pumpkin, tomato, cabbage, Chinese cabbage, edible wild plants, fried vegetables, mandarin orange, fresh fruit juice, and others fruits. Regarding four beverages (coffee, black tea, green tea, and oolong tea), participants were asked to choose one of five categories (almost never, 1 to 2 cups per month, 1 to 2 cups per week, 3 to 4 cups per week, or almost daily). Additionally, if they take these beverages almost daily, they were also asked to report the number of cups. For miso soup, the frequency of consumption (almost never, several times per month, every other day, or almost daily) was asked, and the number of cups per day was obtained if subjects chose the category of ‘almost daily’. Alcohol intake was based on the usual weekly intake of Japanese sake, beer, wine, whiskey and distilled spirits among current drinkers. In the above described foods, data of some food items were not collected in a few study areas because a different questionnaire was used there.

### Data analysis

Proportional hazard model was used to determine hazard ratios (HRs) and their 95% confidence intervals (CIs) of stomach cancer for different levels of the dietary intakes. Analyses were conducted separately for males and females. Statistical significance (two sided) was based on the ratio of the regression coefficient and its standard error. Statistical analysis (PHREG procedure) was performed by the Statistical Analysis System^®^ (SAS institute, 1983).

## RESULTS

During the follow up period, a total of 859 stomach cancer deaths (574 males and 285 females) were obtained. The results of age adjusted analyses are presented in Table 1. There was a non-significant inverse association between the intake of vegetables such as carrots, tomatoes and spinach, and stomach cancer risk in both sexes. There was a weak non-significant association between the intake of cabbage and Chinese cabbage, and mortality among females. The relative risk of fruit consumption such as mandarin orange and pure fruit juice also did not decrease among men and women. In contrast to these results, the consumption of mushroom was associated with the increased risk of stomach cancer in females (HR=1.54, 95% CI: 1.03-2.28).

**Table 1.** Age adjusted hazard ratios of stomach cancer for intake of vegetables, fruit, and other foods by sex.

	Male				Female			
	
	No. of death	Hazard ratio	95% confidence interval	p for trend	No. of death	Hazard ratio	95% confidence interval	p for trend
Spinach								
1-2/month or less	34	1.00	(reference)		10	1.00	(reference)	
1-2/week	121	1.16	0.79 - 1.69		50	1.29	0.65 - 2.55	
3-4/week	119	1.10	0.75 - 1.62		67	1.51	0.78 - 2.93	
1+/day	162	1.29	0.89 - 1.87	0.16	79	1.40	0.72 - 2.70	0.38

Carrot • pumpkin								
1-2/month or less	71	1.00	(reference)		24	1.00	(reference)	
1-2/week	150	1.28	0.96 - 1.69		56	0.86	0.54 - 1.39	
3-4/week	98	1.19	0.88 - 1.61		64	1.04	0.65 - 1.67	
1+/day	59	1.14	0.80 - 1.61	0.60	54	1.15	0.71 - 1.86	0.24

Tomato								
1-2/month or less	145	1.00	(reference)		72	1.00	(reference)	
1-2/week	127	1.05	0.83 - 1.33		52	0.80	0.56 - 1.15	
3-4/week	82	1.06	0.81 - 1.39		43	0.91	0.62 - 1.32	
1+/day	62	1.19	0.88 - 1.61	0.29	44	1.10	0.76 - 1.60	0.66

Cabbage								
1-2/month or less	53	1.00	(reference)		15	1.00	(reference)	
1-2/week	116	0.81	0.59 - 1.12		49	1.01	0.57 - 1.81	
3-4/week	113	0.90	0.65 - 1.25		56	1.16	0.65 - 2.04	
1+/day	111	1.11	0.80 - 1.54	0.15	71	1.49	0.85 - 2.60	0.03

Chinese cabbage								
1-2/month or less	67	1.00	(reference)		33	1.00	(reference)	
1-2/week	122	0.99	0.73 - 1.33		58	1.03	0.67 - 1.58	
3-4/week	86	0.94	0.68 - 1.30		41	0.94	0.60 - 1.49	
1+/day	77	1.18	0.85 - 1.64	0.39	47	1.30	0.83 - 2.03	0.31

Edible wild plants								
None	137	1.00	(reference)		71	1.00	(reference)	
1-2/month	151	1.16	0.92 - 1.46		75	1.26	0.91 - 1.74	
1-2/week	51	1.02	0.74 - 1.41		26	1.05	0.67 - 1.65	
3-4/week or more	48	1.37	0.98 - 1.90	0.16	22	1.22	0.75 - 2.96	0.87

Mandarin orange								
1-2/month or less	94	1.00	(reference)		23	1.00	(reference)	
1-2/week	87	0.77	0.57 - 1.03		33	0.90	0.53 - 1.53	
3-4/week	84	0.79	0.59 - 1.06		43	1.00	0.60 - 1.66	
1+/day	124	0.92	0.71 - 1.21	0.80	93	1.03	0.65 - 1.63	0.65

Pure fruit juice								
None	90	1.00	(reference)		48	1.00	(reference)	
1-2/month	60	0.91	0.66 - 1.26		19	0.68	0.40 - 1.17	
1-2/week	69	0.77	0.56 - 1.06		38	1.03	0.67 - 1.58	
3-4/week	54	0.85	0.61 - 1.20		34	1.20	0.77 - 1.86	
1+/day	67	1.23	0.89 - 1.68	0.51	31	0.95	0.60 - 1.49	0.59

Bean curd								
1-2/month or less	31	1.00	(reference)		11	1.00	(reference)	
1-2/week	140	1.12	0.76 - 1.65		60	1.24	0.65 - 2.36	
3-4/week	162	1.09	0.74 - 1.60		88	1.38	0.74 - 2.59	
1+/day	139	1.07	0.73 - 1.58	0.97	85	1.41	0.75 - 2.64	0.25

Mushroom								
1-2/month or less	152	1.00	(reference)		46	1.00	(reference)	
1-2/week	123	0.88	0.69 - 1.11		67	1.15	0.79 - 1.67	
3-4/week	66	0.97	0.72 - 1.29		53	1.54	1.03 - 2.28	
1+/day	26	0.89	0.59 - 1.35	0.57	17	1.11	0.64 - 1.94	0.16

The consumption of high salt foods such as miso soup, pickles, and dried fish was not significantly associated with the mortality of stomach cancer in males (Table 2). The relative risks of these foods were close to one. In females, the intake of pickles, fish boiled in soy sauce and strong taste of salty food elevated the risk of stomach cancer, but not significantly.

**Table 2.** Age-adjusted hazard ratios of stomach cancer for intake of salty foods by sex.

	Male				Female			
	
	No. of death	Hazard ratio	95% confidence interval	p for trend	No. of death	Hazard ratio	95% confidence interval	p for trend
Pickles								
1-2/month or less	52	1.00	(reference)		18	1.00	(reference)	
1-2/week	60	1.04	0.72 - 1.51		30	1.56	0.87 - 2.81	
3-4/week	74	1.00	0.70 - 1.42		32	1.32	0.74 - 2.36	
1+/day	330	1.09	0.82 - 1.47	0.48	175	1.47	0.90 - 2.39	0.26

Fish boiled in soy sauce								
None	96	1.00	(reference)		46	1.00	(reference)	
1-2/month	100	0.86	0.65 - 1.13		46	0.99	0.66 - 1.49	
1-2/week	104	0.88	0.67 - 1.17		50	1.09	0.73 - 1.63	
3-4/week	66	1.08	0.79 - 1.48		25	0.99	0.61 - 1.61	
1+/day	30	0.92	0.61 - 1.38	0.87	24	1.57	0.95 - 2.57	0.18

Boiled beans								
None	80	1.00	(reference)		29	1.00	(reference)	
1-2/month	141	0.86	0.65 - 1.13		70	0.84	0.54 - 1.29	
1-2/week	93	0.77	0.57 - 1.05		48	0.79	0.50 - 1.26	
3-4/week	73	1.19	0.86 - 1.63		34	0.97	0.59 - 1.60	
1+/day	27	0.93	0.60 - 1.44	0.61	16	0.84	0.46 - 1.56	0.88

Dried or salty fish								
None	36	1.00	(reference)		29	1.00	(reference)	
1-2/month	83	0.89	0.60 - 1.32		39	0.64	0.40 - 1.04	
1-2/week	141	0.93	0.65 - 1.34		63	0.66	0.42 - 1.02	
3-4/week	66	0.85	0.57 - 1.28		33	0.69	0.42 - 1.13	
1+/day	48	1.14	0.74 - 1.76	0.64	24	0.92	0.53 - 1.58	0.86

Miso soup								
None	15	1.00	(reference)		12	1.00	(reference)	
Several times/month	43	1.42	0.79 - 2.56		25	1.38	0.69 - 2.74	
Several times/week	57	1.50	0.85 - 2.65		28	1.18	0.60 - 2.31	
Everyday	415	1.44	0.86 - 2.42	0.36	201	1.46	0.81 - 2.61	0.19

Miso soup(cup)								
less than 1/day	115	1.00	(reference)		65	1.00	(reference)	
1/day	93	0.90	0.69 - 1.19		65	1.21	0.86 - 1.72	
2/day	114	0.99	0.76 - 1.28		62	1.24	0.87 - 1.77	
3+/day	140	1.17	0.91 - 1.50	0.16	41	1.24	0.84 - 1.84	0.23

Preference for salty foods								
No	9	1.00	(reference)		4	1.00	(reference)	
A little	39	0.90	0.43 - 1.85		30	1.56	0.55 - 4.42	
Fair	210	1.14	0.58 - 2.21		115	1.80	0.66 - 4.88	
Pretty	119	1.11	0.56 - 2.18		35	1.51	0.54 - 4.26	
Very much	51	1.36	0.67 - 2.78	0.12	13	1.89	0.62 - 5.79	0.57

Table 3 shows the relationship between intake of meat, fish and marine foods, and stomach cancer. In males, those who had beef once or twice a week showed a significant decreased risk compared to those who hardly ever consumed beef (HR=0.73, 95% CI: 0.55-0.96). Regarding the intake of chicken, the relative risk of eating it once or twice a month was also significantly reduced (HR=0.62, 95% CI: 0.45-0.86). However, the association of meat, which included sausage and liver, with stomach cancer was not observed in males. On the other hand, those who consumed liver three to four times per week and more than once a day had a increased risk among females, respectively (RR=2.02,95% CI:1.12-3.63, RR=3.16,95% CI:1.16-8.62). The non-significantly higher risk for stomach cancer was found in the third highest consumption of fish (HR=1.62, 95% CI: 0.95-2.75). No clear association was found between the intake of other meat and fish and the risk of stomach cancer in females.

**Table 3.** Age-adjusted hazard ratios of stomach cancer for intake of meat, fish, and marine foods by sex.

	Male				Female			
	
	No. of death	Hazard ratio	95% confidence interval	p for trend	No. of death	Hazard ratio	95% confidence interval	p for trend
Beef								
None	100	1.00	(reference)		48	1.00	(reference)	
1-2/month	119	0.79	0.61 - 1.04		52	1.02	0.69 - 1.51	
1-2/week	92	0.73	0.55 - 0.96		51	1.09	0.74 - 1.62	
3-4/week	34	1.00	0.67 - 1.47		13	0.92	0.50 - 1.69	
1+/day	6	0.90	0.39 - 2.05	0.30	4	2.05	0.74 - 5.68	0.60

Pork								
None	40	1.00	(reference)		26	1.00	(reference)	
1-2/month	84	0.96	0.66 - 1.41		40	1.13	0.69 - 1.85	
1-2/week	173	1.10	0.78 - 1.55		78	1.16	0.74 - 1.82	
3-4/week	66	1.10	0.74 - 1.62		34	1.35	0.81 - 2.26	
1+/day	14	1.05	0.57 - 1.93	0.45	7	1.50	0.65 - 3.48	0.21

Sausage								
None	108	1.00	(reference)		54	1.00	(reference)	
1-2/month	101	0.93	0.71 - 1.22		54	1.31	0.90 - 1.91	
1-2/week	139	1.00	0.77 - 1.28		62	1.13	0.78 - 1.64	
3-4/week	71	1.24	0.92 - 1.67		26	1.18	0.74 - 1.89	
1+/day	20	1.36	0.85 - 2.20	0.10	9	1.82	0.90 - 3.70	0.27

Chicken								
None	54	1.00	(reference)		18	1.00	(reference)	
1-2/month	99	0.62	0.45 - 0.86		50	1.12	0.66 - 1.93	
1-2/week	179	0.71	0.52 - 0.96		92	1.09	0.66 - 1.81	
3-4/week	81	0.84	0.59 - 1.18		46	1.30	0.76 - 2.25	
1+/day	12	0.73	0.39 - 1.37	0.98	7	1.33	0.55 - 3.17	0.33

Liver								
None	173	1.00	(reference)		81	1.00	(reference)	
1-2/month	114	0.84	0.66 - 1.06		56	1.26	0.90 - 1.78	
1-2/week	67	1.06	0.80 - 1.41		32	1.39	0.92 - 2.11	
3-4/week	22	1.27	0.82 - 1.99		13	2.02	1.12 - 3.63	
1+/day	2	0.56	0.14 - 2.28	0.75	4	3.16	1.16 - 8.62	0.00

Fish								
1-2/month or less	45	1.00	(reference)		16	1.00	(reference)	
1-2/week	144	0.85	0.61 - 1.19		65	1.22	0.71 - 2.11	
3-4/week	154	0.90	0.65 - 1.26		94	1.62	0.95 - 2.75	
1+/day	136	0.95	0.68 - 1.33	0.73	63	1.41	0.82 - 2.45	0.13

Boiled fish paste								
None	75	1.00	(reference)		32	1.00	(reference)	
1-2/month	107	1.03	0.77 - 1.38		43	0.91	0.58 - 1.44	
1-2/week	116	1.27	0.95 - 1.69		70	1.47	0.97 - 2.24	
3-4/week or more	58	1.21	0.86 - 1.71	0.10	30	1.25	0.76 - 2.06	0.06

Seaweed								
1-2/month or less	59	1.00	(reference)		22	1.00	(reference)	
1-2/week	133	0.90	0.66 - 1.22		53	0.75	0.46 - 1.24	
3-4/week	176	1.15	0.86 - 1.55		79	0.90	0.56 - 1.44	
1+/day	158	1.04	0.77 - 1.40	0.29	103	0.97	0.61 - 1.54	0.38

Men who had consumed yogurt once or twice a month showed a significant decreased risk compared to those who had hardly ever consumed yogurt (HR=0.69, 95% CI: 0.49-0.98) (Table 4). Daily products such as eggs and milk were not associated with stomach cancer in males. The relative risks for the second to quintile were close to null value. For intake of eggs among females, the relative risks for third and quartile significantly increased (HR=2.11, 95% CI: 1.09-4.09, HR=2.32, 95% CI: 1.22-4.42, respectively). Meantime, butter intake had a non-significant inverse association with stomach cancer risk (HR=0.37, 95% CI: 0.14-1.01).

**Table 4.** Age adjusted hazard ratios of stomach cancer for intake of daily products by sex.

	Male				Female			
	
	No. of death	Hazard ratio	95% confidence interval	p for trend	No. of death	Hazard ratio	95% confidence interval	p for trend
Egg								
1-2/month or less	34	1.00	(reference)		10	1.00	(reference)	
1-2/week	119	1.16	0.79 - 1.69		52	1.80	0.91 - 3.53	
3-4/week	118	0.90	0.62 - 1.32		76	2.11	1.09 - 4.09	
1+/day	260	1.13	0.79 - 1.62	0.64	124	2.32	1.22 - 4.42	0.01

Milk								
None	100	1.00	(reference)		60	1.00	(reference)	
1-2/month	41	1.13	0.79 - 1.63		11	0.62	0.33 - 1.18	
1-2/week	75	1.17	0.87 - 1.58		32	0.90	0.58 - 1.38	
3-4/week	66	1.08	0.79 - 1.48		30	0.78	0.50 - 1.21	
1+/day	223	1.06	0.84 - 1.35	0.81	115	0.83	0.60 - 1.13	0.34

Yogurt								
None	247	1.00	(reference)		100	1.00	(reference)	
1-2/month	35	0.69	0.49 - 0.98		29	0.95	0.63 - 1.43	
1-2/week	28	0.86	0.58 - 1.28		33	1.38	0.93 - 2.05	
3-4/week	18	1.22	0.76 - 1.97		10	0.85	0.44 - 1.63	
1+/day	16	0.82	0.50 - 1.37	0.47	11	0.88	0.47 - 1.64	0.93

Cheese								
None	191	1.00	(reference)		106	1.00	(reference)	
1-2/month	82	0.93	0.72 - 1.20		47	1.31	0.92 - 1.85	
1-2/week	51	1.08	0.80 - 1.48		19	0.93	0.57 - 1.52	
3-4/week	23	1.32	0.86 - 2.04		5	0.60	0.24 - 1.46	
1+/day	8	0.79	0.39 - 1.61	0.64	6	1.18	0.52 - 2.69	0.80

Butter								
None	190	1.00	(reference)		104	1.00	(reference)	
1-2/month	78	0.97	0.75 - 1.27		27	0.76	0.50 - 1.17	
1-2/week	47	0.96	0.70 - 1.33		31	1.27	0.85 - 1.90	
3-4/week	19	0.92	0.57 - 1.47		4	0.37	0.14 - 1.01	
1+/day	11	0.70	0.38 - 1.29	0.33	11	1.22	0.65 - 2.26	0.89

Margarine								
None	195	1.00	(reference)		89	1.00	(reference)	
1-2/month	54	0.83	0.61 - 1.12		29	0.98	0.64 - 1.50	
1-2/week	71	1.14	0.87 - 1.49		39	1.09	0.74 - 1.59	
3-4/week	29	0.92	0.62 - 1.36		14	0.69	0.39 - 1.22	
1+/day	25	0.72	0.48 - 1.10	0.36	20	0.82	0.50 - 1.33	0.30

Table 5 presented the relative risk of stomach cancer for starch food and style of breakfast. No significant association of rice intake with stomach cancer at present or in the thirties was observed in both males and females. Regarding the intake of potatoes once or twice a week in females, the relative risk significantly decreased (HR=0.66, 95% CI: 0.44-0.98). A western style breakfast showed a preventive association with stomach cancer in males (HR=0.49, 95% CI: 0.35-0.70). In contrast, tea gruel significantly elevated the risk of stomach cancer in males (HR=1.50, 95% CI: 1.03-2.18).

**Table 5.** Age adjusted hazard ratios of stomach cancer for intake of starch food and style of breakfast by sex.

	Male				Female			
	
	No. of death	Hazard ratio	95% confidence interval	p for trend	No. of death	Hazard ratio	95% confidence interval	p for trend
Rice (cup)								
1/day or less	47	0.93	0.68 - 1.28		36	1.25	0.86 - 1.80	
2/day	96	1.09	0.85 - 1.39		52	0.96	0.70 - 1.32	
3/day	198	1.00	(reference)		132	1.00	(reference)	
4/day	60	0.93	0.69 - 1.24		18	0.96	0.58 - 1.57	
5/day	69	1.05	0.80 - 1.38		18	1.31	0.80 - 2.15	
6+/day	69	0.97	0.74 - 1.28	0.87	8	0.75	0.37 - 1.54	0.54

Rice (cup) (thirties)								
1/day or less	7	1.38	0.63 - 3.01		8	1.91	0.89 - 4.08	
2/day	36	0.87	0.58 - 1.32		26	1.09	0.66 - 1.79	
3/day	59	1.00	(reference)		39	1.00	(reference)	
4/day	35	1.15	0.76 - 1.75		23	1.21	0.72 - 2.03	
5/day	54	1.09	0.75 - 1.57		26	0.96	0.58 - 1.58	
6+/day	215	1.08	0.81 - 1.44	0.42	87	1.18	0.80 - 1.72	0.99

Potato								
1-2/month or less	96	1.00	(reference)		36	1.00	(reference)	
1-2/week	181	1.08	0.84 - 1.38		67	0.66	0.44 - 0.98	
3-4/week	146	1.06	0.82 - 1.38		91	0.85	0.58 - 1.26	
1+/day	94	1.08	0.81 - 1.44	0.65	61	0.86	0.57 - 1.29	0.74

Japanese style breakfast								
No	55	1.00	(reference)		36	1.00	(reference)	
Yes	397	1.30	0.98 - 1.72		184	1.24	0.87 - 1.77	

Western style breakfast								
No	418	1.00	(reference)		182	1.00	(reference)	
Yes	34	0.49	0.35 - 0.70		38	0.82	0.58 - 1.17	

Tea gruel for breakfast								
No	396	1.00	(reference)		198	1.00	(reference)	
Yes	29	1.50	1.03 - 2.18		5	0.50	0.21 - 1.22	

Coffee consumption showed a significant inverse association with the risk of stomach cancer in age-adjusted analysis among males, with a relative risk of 0.81 for the fifth intake category (more than one cup per day) (Table 6). However, the relative risk for the consumption of three or four cups of coffee per day was statistical significant in comparison with non-drinkers in females (HR=1.70, 95% CI: 1.09-2.67). Compared to those who drank less than two cups of coffee per week, women who drank three cups or more of coffee per week had an increased risk of stomach cancer(HR=1.66, 95% CI: 1.09-2.54). There was no clear association between levels of consumption of coffee and the risk of stomach cancer among male.

**Table 6.** Age adjusted hazard ratios of stomach cancer for intake of coffee and tea by sex.

	Male				Female			
	
	No. of death	Hazard ratio	95% confidence interval	p for trend	No. of death	Hazard ratio	95% confidence interval	p for trend
Coffee (cup)								
None	151	1.00	(reference)		74	1.00	(reference)	
1-2/month	33	0.82	0.57 - 1.20		17	1.04	0.62 - 1.77	
1-2/week	84	0.92	0.70 - 1.20		36	0.99	0.66 - 1.48	
3-4/week	46	1.00	0.72 - 1.40		26	1.70	1.09 - 2.67	
1+/day	222	0.81	0.65 - 0.99	0.07	105	1.00	0.74 - 1.35	0.81

Coffee (cup)								
2/week or less	268	1.00	(reference)		127	1.00	(reference)	
less than 1/day	46	1.05	0.77 - 1.44		26	1.66	1.09 - 2.54	
1/day	45	0.82	0.60 - 1.12		30	1.03	0.69 - 1.54	
2/day	22	0.55	0.36 - 0.86		16	1.09	0.64 - 1.84	
3+/day	26	1.00	0.66 - 1.50	0.06	3	0.47	0.15 - 1.50	0.79

Black tea (cup)								
None	308	1.00	(reference)		154	1.00	(reference)	
1-2/month	48	0.98	0.72 - 1.33		20	0.75	0.47 - 1.20	
1-2/week	20	0.75	0.48 - 1.18		13	0.82	0.46 - 1.44	
3-4/week or more	32	1.41	0.98 - 2.03	0.29	14	1.03	0.60 - 1.79	0.84

China tea								
less than 1/day	350	1.00	(reference)		174	1.00	(reference)	
1+/day	11	0.70	0.38 - 1.27	0.72	8	0.58	0.28 - 1.17	0.18

## DISCUSSION

In this study, we found no association between stomach cancer mortality and the consumption of fruits and vegetables. The protective effect against stomach cancer of the consumption of a wide range of fruits and vegetables has been examined in epidemiological studies. Many case control studies have shown a decreased risk with fruits and vegetables,^5^^-^^8^ although there are exceptions.^11^^,^^18^ This protective effect might be due to food rich in vitamin C, such as oranges, tomatoes, citrus fruits. Vitamin C can prevent the formation of N-nitroso compounds.^19^^,^^20^ In some studies, dietary intakes of micronutrients have been estimated. These studies have shown a consistent negative association of dietary intakes of vitamin C and carotenoids with stomach cancer.^21^^-^^23^ In contrast, a negative association between the intakes of vegetables and fruits and stomach cancer has been less pronounced in limited cohort studies.^24^ In some cohort studies, the intakes of micronutrients such as vitamin C, *β*-carotene, and other carotenes have been examined.^16^^,^^25^^,^^26^ There was no significant difference in any micronutrients between men developing stomach cancer and those without stomach cancer. A meta-analytic approach to examine the evidence from cohort studies on total vegetable and fruit intake showed no significant protective effects.^27^ In the meantime, an intervention trial using supplementation with *β*-carotene, vitamin E, and selenium showed reductions in stomach cancer mortality.^28^ These results are difficult to generalize for populations who do not take supplements everyday.^24^ In this study, consumption of liver was related to the high risk of stomach cancer in females. The reason for this association might be that women who often consume liver have suffered from anemia due to a stomach disorder including stomach cancer.

No salty food such as miso soup, pickled vegetable, and salted or dried fish was associated with the risk of stomach cancer. It has been suggested that salt intake is strongly associated with intestinal metaplasia and potentiates the effects of carcinogens.^29^ In support of this hypothesis, several epidemiological studies have reported significant association between salt intake or salt attitude and stomach cancer,^10^^,^^12^^,^^22^ but these are not universal findings.^25^^,^^30^^,^^31^ Part of the reason for these discrepant results is that the intake of sodium is difficult to quantify.^4^ To obtain a valid estimation of salt intake, rigorous dietary intake methodology, including information on discretionary use of table salt and soy sauce or direct measures of salt metabolism should be used. In this study, a food frequency questionnaire without portion size was used. Therefore, the relation between salt intake and stomach cancer risk may not be adequately appraised by this method.

Previous case control studies have not found any significant associations between the consumption of coffee with stomach cancer risk.^7^^,^^31^^-^^34^ Coffee consumption showed a significant inverse association with the risk of stomach cancer in males. Men who had a western style breakfast have a significant lower risk of stomach cancer. Coffee consumption is considered to be a westernized dietary habit. Mutagenic substances have been found in coffee,^35^^-^^37^ although caffeine is not thought to be carcinogenic in humans when consumed in normal amounts.^7^ However, we failed to observe a clear dose-response relationship between coffee consumption and stomach cancer risk. In addition, the intake of coffee increased the risk of stomach cancer in females. Therefore, this contradictious association between coffee consumption and stomach cancer in males and females needs further investigation.

Intakes of beef, chicken and yogurt in males, and potatoes in females showed a significantly decreased risk. However, this trend did not indicate a significant association. Frequencies of intakes of these foods were rather low, once or twice per week or month. These results suggested the necessity of further epidemiological studies to verify the association between these foods and risk of stomach cancer.

It is not certain why the present study did not find a significant association between the intake of foods and stomach cancer, as shown in the past cohort studies. The possibility exists that the food frequency questionnaire used in this study was not sensitive enough to detect meaningful differences in dietary intake due to the small number of food items (33 foods). The present study did not specifically include a number of other food items that have been positively related to stomach cancer in past studies, such as foods rich in nitrate-related compounds. We were not able to adjust for total calories, because a quantitative estimate of food consumption was not available. In addition, even if some foods showed the association with stomach cancer risk in this study, most of the association did not indicate a dose-response relationship. Therefore, it is important to carefully consider the results.

We have studied the association between the consumption of different foods and stomach cancer deaths. Subjects with subclinical stomach cancer may have changed their diet because of symptoms. We should also consider the possibility that the results of the present study based on mortality data reflected not causal factors, but prognostic factors of stomach cancer.

In conclusion, no clear association with individual food consumption was observed, although the results from this prospective study indicate that some foods were associated with stomach cancer risk. We also collected incidence cases of stomach cancer in this cohort. Further study will be required to use these data.

## MEMBER LIST OF THE JACC STUDY GROUP

The present investigators involved, with the co-authorship of this paper, in the JACC Study and their affiliations are as follows: Dr. Akiko Tamakoshi (present chairman of the study group), Nagoya University Graduate School of Medicine; Dr. Mitsuru Mori, Sapporo Medical University School of Medicine; Dr. Yutaka Motohashi, Akita University School of Medicine; Dr. Ichiro Tsuji, Tohoku University Graduate School of Medicine; Dr. Yosikazu Nakamura, Jichi Medical School; Dr. Hiroyasu Iso, Institute of Community Medicine, University of Tsukuba; Dr. Haruo Mikami, Chiba Cancer Center; Dr. Yutaka Inaba, Juntendo University School of Medicine; Dr. Yoshiharu Hoshiyama, University of Human Arts and Sciences; Dr. Hiroshi Suzuki, Niigata University School of Medicine; Dr. Hiroyuki Shimizu, Gifu University School of Medicine; Dr. Hideaki Toyoshima, Nagoya University Graduate School of Medicine; Dr. Kenji Wakai, Aichi Cancer Center Research Institute; Dr. Shinkan Tokudome, Nagoya City University Graduate School of Medical Sciences; Dr. Yoshinori Ito, Fujita Health University School of Health Sciences; Dr. Shuji Hashimoto, Fujita Health University School of Medicine; Dr. Shogo Kikuchi, Aichi Medical University School of Medicine; Dr. Akio Koizumi, Graduate School of Medicine and Faculty of Medicine, Kyoto University; Dr. Takashi Kawamura, Kyoto University Center for Student Health; Dr. Yoshiyuki Watanabe, Kyoto Prefectural University of Medicine Graduate School of Medical Science; Dr. Tsuneharu Miki, Graduate School of Medical Science, Kyoto Prefectural University of Medicine; Dr. Chigusa Date, Faculty of Human Environmental Sciences, Mukogawa Women’s University ; Dr. Kiyomi Sakata, Wakayama Medical University; Dr. Takayuki Nose, Tottori University Faculty of Medicine; Dr. Norihiko Hayakawa, Research Institute for Radiation Biology and Medicine, Hiroshima University; Dr. Takesumi Yoshimura, Fukuoka Institute of Health and Environmental Sciences; Dr. Akira Shibata, Kurume University School of Medicine; Dr. Naoyuki Okamoto, Kanagawa Cancer Center; Dr. Hideo Shio, Moriyama Municipal Hospital; Dr. Yoshiyuki Ohno, Asahi Rosai Hospital; Dr. Tomoyuki Kitagawa, Cancer Institute of the Japanese Foundation for Cancer Research; Dr. Toshio Kuroki, Gifu University; and Dr. Kazuo Tajima, Aichi Cancer Center Research Institute.
